# Osteoporosis imaging: effects of bone preservation on MDCT-based trabecular bone microstructure parameters and finite element models

**DOI:** 10.1186/s12880-015-0066-z

**Published:** 2015-06-26

**Authors:** Thomas Baum, Eduardo Grande Garcia, Rainer Burgkart, Olga Gordijenko, Hans Liebl, Pia M. Jungmann, Michael Gruber, Tina Zahel, Ernst J. Rummeny, Simone Waldt, Jan S. Bauer

**Affiliations:** Institut für Radiologie, Klinikum rechts der Isar, Technische Universität München, Ismaninger Str. 22, 81675 München, Germany; Klinik für Orthopädie, Abteilung für Biomechanik, Klinikum rechts der Isar, Technische Universität München, Ismaninger Str. 22, 81675 München, Germany; Universitätsklinik für Radiologie und Nuklearmedizin, Abteilung für Neuroradiologie und Muskuloskeletale Radiologie, Medizinischen Universität Wien, Währinger Gürtel 18-20, 1090 Wien, Austria; Abteilung für Neuroradiologie, Klinikum rechts der Isar, Technische Universität München, Ismaninger Str. 22, 81675 München, Germany

**Keywords:** Osteoporosis, Bone preservation, Trabecular bone microstructure, Finite element model

## Abstract

**Background:**

Osteoporosis is defined as a skeletal disorder characterized by compromised bone strength due to a reduction of bone mass and deterioration of bone microstructure predisposing an individual to an increased risk of fracture. Trabecular bone microstructure analysis and finite element models (FEM) have shown to improve the prediction of bone strength beyond bone mineral density (BMD) measurements. These computational methods have been developed and validated in specimens preserved in formalin solution or by freezing. However, little is known about the effects of preservation on trabecular bone microstructure and FEM. The purpose of this observational study was to investigate the effects of preservation on trabecular bone microstructure and FEM in human vertebrae.

**Methods:**

Four thoracic vertebrae were harvested from each of three fresh human cadavers (*n* = 12). Multi-detector computed tomography (MDCT) images were obtained at baseline, 3 and 6 month follow-up. In the intervals between MDCT imaging, two vertebrae from each donor were formalin-fixed and frozen, respectively. BMD, trabecular bone microstructure parameters (histomorphometry and fractal dimension), and FEM-based apparent compressive modulus (ACM) were determined in the MDCT images and validated by mechanical testing to failure of the vertebrae after 6 months.

**Results:**

Changes of BMD, trabecular bone microstructure parameters, and FEM-based ACM in formalin-fixed and frozen vertebrae over 6 months ranged between 1.0–5.6 % and 1.3–6.1 %, respectively, and were not statistically significant (*p* > 0.05). BMD, trabecular bone microstructure parameters, and FEM-based ACM as assessed at baseline, 3 and 6 month follow-up correlated significantly with mechanically determined failure load (r = 0.89–0.99; *p* < 0.05). The correlation coefficients r were not significantly different for the two preservation methods (*p* > 0.05).

**Conclusions:**

Formalin fixation and freezing up to six months showed no significant effects on trabecular bone microstructure and FEM-based ACM in human vertebrae and may both be used in corresponding in-vitro experiments in the context of osteoporosis.

## Background

Osteoporosis is defined as a skeletal disorder characterized by compromised bone strength due to a reduction of bone mass and deterioration of bone microstructure predisposing an individual to an increased risk of fracture [1]. Osteoporotic vertebral and hip fractures are associated with an increased mortality [2]. Due to the aging population, the prevalence of osteoporosis and consecutively the incidence of osteoporotic fractures is expected to increase [3]. Therefore, osteoporosis is classified as public health problem.

The World Health Organisation (WHO) based the diagnosis of osteoporosis on the measurement of bone mineral density (BMD) at the spine and hip using dual-energy X-ray absorptiometry (DXA) [4]. Alternatively, BMD can be assessed by using quantitative computed tomography (QCT). QCT allows for the assessment of volumetric BMD (vBMD), in contrast to DXA assessing areal BMD (aBMD) [5]. Importantly, BMD values often underestimate fracture risk, since osteoporotic fractures frequently occur in patients with non-pathological BMD values [6, 7]. Therefore, considerable research effort has been undertaken to improve fracture risk prediction by using high-resolution imaging techniques including high-resolution peripheral quantitative computed tomography (hr-pQCT), multi-detector computed tomography (MDCT), and magnetic resonance imaging (MRI) [8]. Trabecular bone microstructure parameters and finite element models (FEM) have been computed in the acquired images which significantly improved prediction of bone strength beyond BMD [9]. These computational methods have been developed in human specimens in-vitro and validated with mechanical bone strength measurements as gold standard.

After harvesting, the specimens are usually preserved in formalin solution or are frozen until imaging and mechanical testing is performed. Previous studies have reported that human cortical and trabecular bone samples showed no significant differences in their mechanical properties and tissue parameters (including mineral and lipid content and composition) after freezing or formalin fixation up to several weeks [10, 11]. However, weakened viscoelastic and plastic properties of bovine, murine, and human bone by formalin fixation up to six months were demonstrated as compared to freezing [12–14]. Lochmüller et al. reported that DXA-based BMD measurements in human cadavers within 48 h of death and after 10 months of formalin fixation were not significantly different [15].

Little is known about the effects of preservation on trabecular bone microstructure and FEM in human bone specimens. Therefore, the purpose of our study was to investigate the effects of preservation (formalin fixation and freezing) on trabecular bone microstructure and FEM in intact, human vertebrae as determined by MDCT imaging at baseline, 3 and 6 month follow-up and validated by mechanical testing to failure of the vertebrae after 6 months.

## Methods

### Specimens

Donors with a history of pathological bone changes other than osteoporosis (i.e., bone metastases, hematological, or metabolic bone disorders) were excluded at the outset. Four thoracic vertebrae between the thoracic vertebra 5 and 12 were harvested from each of three fresh human cadavers (*n* = 12). The donors consisted of one osteoporotic woman aged 74 years and two non-osteoporotic men aged 46 and 62 years, respectively. They had dedicated their body for educational and research purposes to the local Institute of Anatomy in compliance with local institutional and legislative requirements. The study protocol was reviewed and approved by the local Institutional Review Boards (Ethikkommission der Fakultaet fuer Medizin der Technischen Universitaet Muenchen). The surrounding muscle, fat tissue, and intervertebral discs were completely removed from the vertebrae. Each vertebra was embedded in resin (Rencast Isocyanat and Polyol, Huntsman Group, Bad Säckingen, Germany) up to 2 mm above respectively below their vertebral endplates for the purpose of mechanical testing. The resin fixation was performed with parallel alignment of the upper and lower endplate of the vertebrae with the outer surface of the resin chock to guarantee strict axial loading conditions of the vertebrae during the uniaxial mechanical test.

MDCT imaging was performed at baseline, 3 and 6 month follow-up. In the intervals between the MDCT acquisitions, two vertebrae from each donor were stored in a 3.5 % formalin solution, while the other two vertebrae were stored in sealed plastic bags in a freezer at −40 °C. The vertebrae in the freezer were defrosted for 18 h at 20 °C before 3 and 6 month follow-up MDCT imaging, respectively. All vertebrae were degassed in sodium chloride solution at least 3 h before MDCT imaging to prevent air artifacts. The vertebrae were sealed in vacuum plastic boxes filled with sodium chloride solution during MDCT imaging.

### Imaging

MDCT images of the vertebrae at baseline, 3 and 6 month follow-up were acquired by using a clinical whole-body 256-row CT scanner (iCT, Philips Medical Care, Best, Netherlands). Scan parameters were a tube voltage of 120 kVp, a tube load of 585 mAs, an image matrix of 1024 × 1024 pixels, and a field of view of 150 mm. Transverse sections were reconstructed with a high-resolution bone kernel (YE). The interpolated voxel size was of 146 × 146 × 300 μm^3^, while the real spatial resolution, as determined at ρ50 of the modulation-transfer-function, was 250 × 250 × 600 μm^3^. A dedicated calibration phantom (Mindways Osteoporosis Phantom, San Francisco, CA, USA) was placed in the scanner mat beneath the vertebrae.

### Assessment of BMD and trabecular bone microstructure

**Fig. 1:**
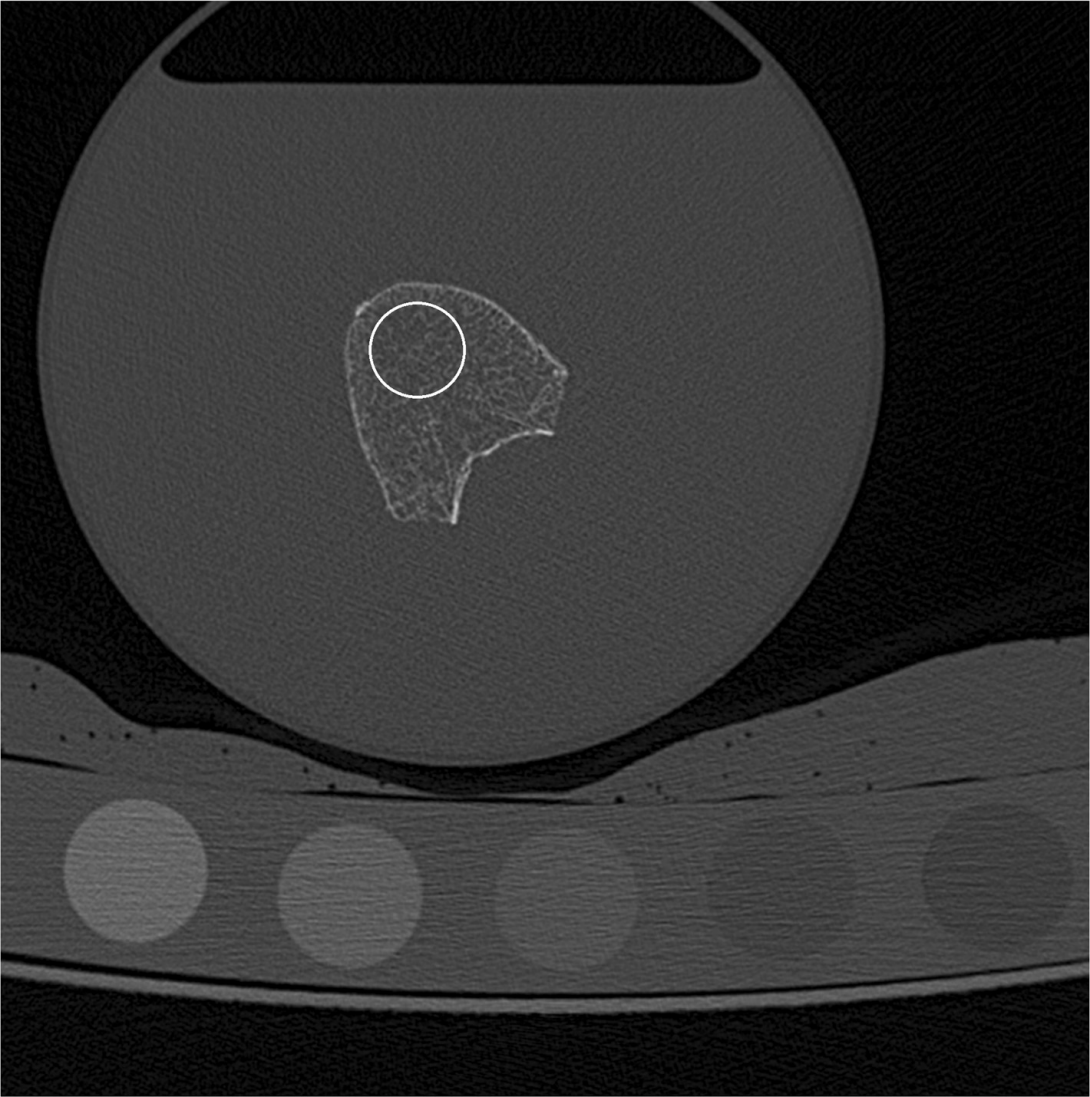
Representative MDCT image of a vertebra: a circular region of interest (white) was placed in the ventral half of the vertebral body in the 15 most central slices equidistant to its endplates. The calibration phantom was positioned below the plastic box containing the vertebrae

MDCT images obtained at baseline, 3 and 6 month follow-up were transferred to a remote LINUX workstation and loaded into an in-house developed program based on IDL (Interactive Data Language, Research Systems, Bolder, CO, USA). Firstly, the 15 most central slices displaying the vertebra equidistant to its endplates were identified. Then, 15 circular regions of interest (ROIs) were manually placed in the ventral half of the vertebral body in the selected slices of the MDCT images similar to QCT-based BMD measurements [5]. The circular ROIs had a diameter of 10 mm (Fig. 1). ROIs’ pixel attenuations in [HU; Hounsfield Units] were converted into BMD values in [mg/cm^3^ calcium hydroxyapatite] by using the calibration phantom. Afterwards, MDCT images were binarized to calculate trabecular bone microstructure parameters. An optimized global threshold was applied to all MDCT images. Similar to previous studies, 200 mg/cm^3^ calcium hydroxyapatite was identified as optimized global threshold [16, 17]. Four morphometric parameters were calculated in the ROIs in analogy to standard histomorphometry using the mean intercept length method [18]: bone volume divided by total volume (BV/TV), trabecular number (TbN; [mm^−1^]), trabecular separation (TbSp; [mm]), and trabecular thickness (TbTh; [mm]). Parameters were labeled as apparent (app.) values, since given the limited spatial resolution they cannot depict the true trabecular microstructure. Furthermore, fractal dimension (FD) as texture measurement of the trabecular bone microstructure was determined in the MDCT images using a box counting algorithm as previously described [16]. The reproducibility error expressed as the root mean square error coefficient of variation amounted to 1.2 % for BMD and ranged between 0.5 % and 2.0 % as outlined in a previous study [16].

### FEM

**Fig. 2:**
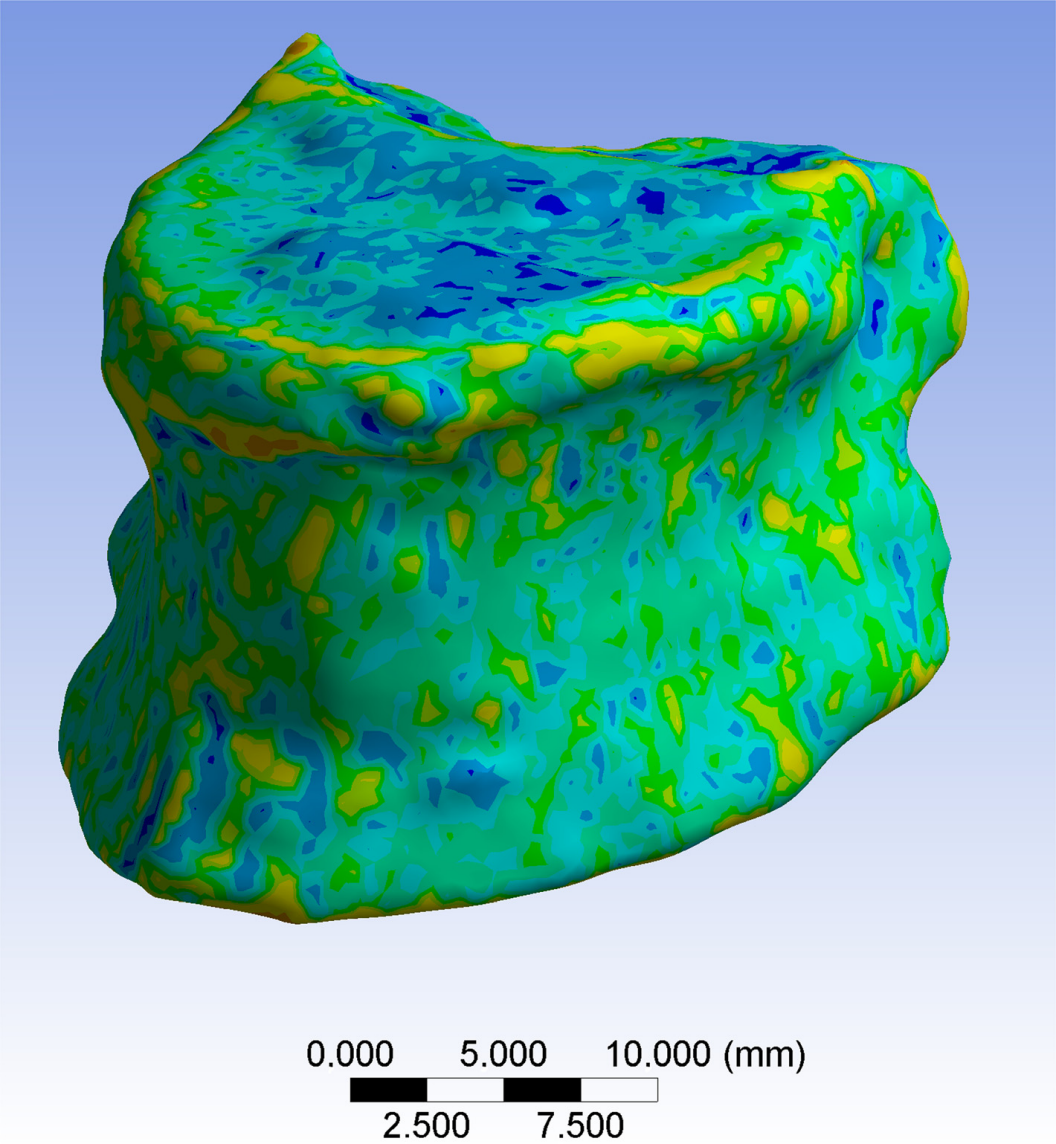
MDCT-based FEM of a representative vertebral body. The BMD distribution is color-coded and used for the assignment of the material properties for each element of the FEM

Finite element models (FEM) were computed in the baseline, 3 and 6 month follow-up MDCT images to assess apparent compressive modulus (ACM) of each vertebral body in the superior-inferior direction. Three-dimensional models of the vertebrae were created from the MDCT images by identifying the contour of the vertebrae. The in-plane MDCT resolution was selected as mesh refinement. The uniform hexahedral meshes were generated by using ANSYS Workbench (ANSYS, Canonsburg, PA, USA). The material properties of each element were assigned by using a mapping procedure. Firstly, the elements’ values in [HU] were converted into BMD values *ρ*_*BMD*_ in [g/cm^3^ calcium hydroxyapatite] by using the calibration phantom. Secondly, the elements’ information (position and *ρ*_*BMD*_) were saved in a text file. Thirdly, a subroutine written in APDL (ANSYS Parametric Design Language) was used to read the text file and assign the material properties to each element (Fig. 2). The equation *ρ*_*ash*_ = 1.22 *ρ*_*BMD*_ + 0.0526 *g*/*cm*3 was used for the conversion of *ρ*_*BMD*_ into *ρ*_*ash*_ [19, 20]. The isotropic elastic modulus E in [N/mm^2^] was determined for each element by using the established relationships between E and *ρ*_*ash*_ as reported previously [21–23]: E = 33900*ρ*_*ash*_^2.20^; *ρ*_*ash*_ ≤ 0.27, E = 5307*ρ*_*ash*_ + 469; 0.27 < *ρ*_*ash*_ < 0.6, and E = 10200 *ρ*_*ash*_^2.01^; *ρ*_*ash*_ ≥ 0.6. Each element was assigned a Poisson’s ratio of *ν* = 0.3 [23]. Finally, the apparent compressive modulus (ACM) of the FEMs in [N/mm^2^] was obtained by applying a displacement force on one vertebral endplate and fixing the opposite one.

### Mechanical testing

After 6 month follow-up MDCT imaging, the resin embedded vertebrae were fixed in a mechanical testing system (Wolpert Werkstoffprüfmaschinen AG, Schaffhausen, Switzerland). The mechanical testing was performed similar to previous studies [16, 24, 25]. Firstly, ten pre-conditioning cycles with uniaxial tension-compression up to a load between 10 N and 400 N with a rate of 5 mm/min were applied. Then, a monotonic, uniaxial compression was performed at the same rate. The load–displacement curve was recorded and vertebral failure load (FL) was defined as the first peak of the load–displacement curve with a subsequent drop of >10 %.

### Statistical analysis

The statistical analyses were performed with SPSS (SPSS, Chicago, IL, USA). All tests were done using a two-sided 0.05 level of significance. Mean and standard deviation (SD) of FEM-based ACM, BMD, and trabecular bone microstructure parameters were calculated at each time point separately for the formalin-fixed and frozen vertebrae. The Kolmogorov-Smirnov test showed for most parameters a significant difference from a normal distribution (*p* < 0.05). Therefore, changes of FEM-based ACM, BMD, and trabecular bone microstructure parameters over 6 months were assessed by using the Friedmann test separately for the formalin-fixed and frozen vertebrae. The root-mean-square coefficients of variation (RMSCV) in [%] were calculated to express the changes of each parameter over time [26]. Correlations between FEM-based ACM, BMD, and trabecular bone microstructure parameters with FL were evaluated with the Spearman’s rank correlation coefficient (r). Significant differences between correlation coefficients were assessed by using the Fisher Z transformation.

## Results

Failure load values of all specimens are shown in Table 1. Mean ± SD of FEM-based ACM, BMD, and trabecular bone microstructure parameters of the formalin-fixed and frozen vertebrae at baseline, 3 and 6 month follow-up are listed in Table 2. Changes of the computed parameters expressed as RMSCV ranged between 1.0–5.6 % and 1.3–6.1 % in formalin-fixed and frozen vertebrae, respectively (Table 2). Neither formalin fixation nor freezing significantly changed the computed parameters over six months (*p* > 0.05; Table 2).

**Table 1:** Failure load values in [N] for each specimen

ID	Preservation	Failure load
Donor 1 vertebra 1	frozen	3181
Donor 1 vertebra 2	formalin	3991
Donor 1 vertebra 3	frozen	3719
Donor 1 vertebra 4	formalin	4147
Donor 2 vertebra 1	frozen	1212
Donor 2 vertebra 2	formalin	1912
Donor 2 vertebra 3	frozen	1704
Donor 2 vertebra 4	formalin	1853
Donor 3 vertebra 1	frozen	1951
Donor 3 vertebra 2	formalin	1990
Donor 3 vertebra 3	frozen	2513
Donor 3 vertebra 4	formalin	3141

**Table 2:** Mean ± SD of FEM-based ACM, BMD, and trabecular bone microstructure parameters of the formalin-fixed and frozen vertebrae at baseline, 3 and 6 month follow-up. Changes of the computed parameters over 6 months are expressed as root-mean-square coefficients of variation (RMSCV) and were not significant (*p* > 0.05) as assessed by using the Friedmann test

	Preservation	Baseline	3 month follow-up	6 month follow-up	RMSCV [%]	p-value
FEM-based ACM [N/mm^2^]	frozen (*n* = 6)	498 ± 214	500 ± 197	504 ± 187	4.0	0.846
FEM-based ACM [N/mm^2^]	formalin (*n* = 6)	494 ± 164	480 ± 152	494 ± 162	2.9	0.513
BMD [mg/cm^3^]	frozen (*n* = 6)	125 ± 34	125 ± 35	124 ± 36	1.3	0.565
BMD [mg/cm^3^]	formalin (*n* = 6)	128 ± 34	129 ± 34	129 ± 34	1.0	0.467
app. BV/TV	frozen (*n* = 6)	0.266 ± 0.121	0.274 ± 0.108	0.277 ± 0.110	5.4	0.311
app. BV/TV	formalin (*n* = 6)	0.269 ± 0.199	0.261 ± 0.123	0.263 ± 0.114	4.0	0.119
app. TbN [mm^−1^]	frozen (*n* = 6)	0.860 ± 0.270	0.864 ± 0.206	0.894 ± 0.248	4.4	0.311
app. TbN [mm^−1^]	formalin (*n* = 6)	0.835 ± 0.255	0.832 ± 0.211	0.847 ± 0.264	4.6	0.846
app. TbSp [mm]	frozen (*n* = 6)	0.990 ± 0.479	0.923 ± 0.367	0.910 ± 0.398	6.1	0.223
app. TbSp [mm]	formalin (*n* = 6)	1.048 ± 0.555	1.035 ± 0.507	1.047 ± 0.559	5.6	0.846
app. TbTh [mm]	frozen (*n* = 6)	0.295 ± 0.050	0.307 ± 0.053	0.301 ± 0.041	3.0	0.119
app. TbTh [mm]	formalin (*n* = 6)	0.308 ± 0.056	0.298 ± 0.069	0.297 ± 0.052	4.0	0.135
FD	frozen (*n* = 6)	1.453 ± 0.145	1.472 ± 0.114	1.484 ± 0.139	2.0	0.311
FD	formalin (*n* = 6)	1.471 ± 0.151	1.470 ± 0.134	1.459 ± 0.159	2.6	0.607

*FEM* finite element model, *ACM* apparent compressive modulus, *BMD* bone mineral density, *app. BV/TV* apparent bone volume divided by total volume, *app. TbN* apparent trabecular number, *app. TbSp* apparent trabecular separation, *app. TbTh* apparent trabecular thickness, *FD* fractal dimension

FEM-based ACM, BMD, and trabecular bone microstructure parameters at baseline, 3 and 6 month follow-up showed significant correlations with FL for both, formalin-fixed and frozen vertebrae (*p* < 0.05; Table 3). Correlations coefficients r ranged between 0.89 and 0.99, and were not significantly different for the two preservation methods as compared by using the Fisher Z transformation (*p* > 0.05).

**Table 3:** Spearman’s rank correlation coefficients (r with respective p-value) of FEM-based ACM, BMD, and trabecular bone microstructure parameters of the formalin-fixed and frozen vertebrae at baseline, 3 and 6 month follow-up with failure load (FL) as determined after six months. Correlation coefficients were not significantly different for the two preservation methods as compared by using the Fisher Z transformation (*p* > 0.05)

			FL [N]	
	Preservation	Baseline	3 month follow-up	6 month follow-up
FEM-based ACM [N/mm^2^]	frozen (*n* = 6)	0.94 (*p* = 0.005)	0.99 (*p* < 0.001)	0.99 (*p* < 0.001)
FEM-based ACM [N/mm^2^]	formalin (*n* = 6)	0.94 (*p* = 0.005)	0.94 (*p* = 0.005)	0.94 (*p* = 0.005)
BMD [mg/cm^3^]	frozen (*n* = 6)	0.99 (*p* < 0.001)	0.99 (*p* < 0.001)	0.99 (*p* < 0.001)
BMD [mg/cm^3^]	formalin (*n* = 6)	0.89 (*p* = 0.019)	0.93 (*p* = 0.008)	0.89 (*p* = 0.019)
app. BV/TV	frozen (*n* = 6)	0.94 (*p* = 0.005)	0.93 (*p* = 0.008)	0.89 (*p* = 0.019)
app. BV/TV	formalin (*n* = 6)	0.94 (*p* = 0.005)	0.89 (*p* = 0.019)	0.89 (*p* = 0.019)
app. TbN [mm^−1^]	frozen (*n* = 6)	0.94 (*p* = 0.005)	0.89 (*p* = 0.019)	0.89 (*p* = 0.019)
app. TbN [mm^−1^]	formalin (*n* = 6)	0.94 (*p* = 0.005)	0.89 (*p* = 0.019)	0.89 (*p* = 0.019)
app. TbSp [mm]	frozen (*n* = 6)	−0.94 (*p* = 0.005)	−0.89 (*p* = 0.019)	−0.89 (*p* = 0.019)
app. TbSp [mm]	formalin (*n* = 6)	−0.94 (*p* = 0.005)	−0.89 (*p* = 0.019)	−0.89 (*p* = 0.019)
app. TbTh [mm]	frozen (*n* = 6)	0.94 (*p* = 0.005)	0.99 (*p* < 0.001)	0.94 (*p* = 0.005)
app. TbTh [mm]	formalin (*n* = 6)	0.94 (*p* = 0.005)	0.94 (*p* = 0.005)	0.89 (*p* = 0.019)
FD	frozen (*n* = 6)	0.94 (*p* = 0.005)	0.94 (*p* = 0.005)	0.93 (*p* = 0.008)
FD	formalin (*n* = 6)	0.89 (*p* = 0.019)	0.89 (*p* = 0.019)	0.89 (*p* = 0.019)

*FEM* finite element model, *ACM* apparent compressive modulus, *BMD* bone mineral density, *app. BV/TV* apparent bone volume divided by total volume, *app. TbN* apparent trabecular number, *app. TbSp* apparent trabecular separation, *app. TbTh* apparent trabecular thickness, *FD* fractal dimension

## Discussion

Human bone specimens have been frequently used to assess mechanical features of new orthopedic implants and to validate new computational methods for the improvement of fracture risk prediction in the context of osteoporosis. Fresh bone specimens would represent the best conditions to guarantee the original structural and mechanical properties. However, the availability of fresh specimens is limited and the setup of many studies requires some type of preservation of the bone tissue due to time constraints. Freezing has the advantages of not significant altering the mechanical properties of human bone specimens [11, 14]. The disadvantage of freezing is the risk of infection of investigators working on bone specimens from a variety of pathogens including HIV and the hepatitis virus [27]. Therefore, bone specimens are often embalmed in formalin solution to minimize the risk of infection. Furthermore, bone specimens available from pathology dissections include high numbers of patients with severe diseases. In contrast, specimens from courses of macroscopic dissections are usually embalmed in formalin solution and could be used to constitute more representative study samples [15]. These cadaver bodies are generally stored for one year or more after they are embalmed. However, formalin fixation may alter the mechanical properties, BMD, and trabecular bone microstructure of the specimens which are particularly important in the context of osteoporosis.

Controversial findings have been reported with regard to the changes of the mechanical properties due to formalin fixation. Haaren et al. reported that long-term preservation by freezing or formalin fixation up to one year did not alter the mechanical properties of cortical bone in goats [28]. In contrast, Wilke et al. reported that formalin fixation strongly influences the mechanical properties of calf spines [29]. Consistently, further studies reported weakened viscoelastic and plastic properties of bovine, murine, and human bone by formalin fixation up to six months as compared to freezing [12–14].

Changes of DXA-based BMD measurements at the lumbar spine and proximal femur due to formalin fixation were assessed by Lochmüller et al. in seven intact human cadavers [15]. They measured BMD within 48 h of death and after 10 months of formalin fixation, and observed no significant deviation in BMD values. Edmondston et al. investigated the correlation of DXA-based BMD and mechanically determined failure load in ten fresh and ten formalin-fixed sheep lumbar spines [30]. The slopes of the regression for BMD and failure load of both groups were not significantly different.

In the line of these studies, we investigated the effects of preservation on QCT-based BMD and advanced computational methods of osteoporosis research, i.e., trabecular bone microstructure parameters and FEM. In consistency with Lochmüller et al., we observed no significant changes of QCT-based BMD over six months for both formalin-fixed and frozen vertebrae [15]. Furthermore, we demonstrated for the first time that trabecular bone microstructure parameters and FEM-based ACM are not significantly altered due to formalin fixation or freezing over six months. Furthermore, the computed parameters correlated well with mechanically determined bone strength independent of the preservation method. These findings are consistent with the association of BMD and failure load in fresh and formalin-fixed sheep lumbar spines as reported by Edmondston et al. [30].

The strength of our study was use of intact, human vertebrae, since this is the most important in-vitro scenario in the context of osteoporosis research. Previous studies have been often limited by investigating trabecular or cortical bone samples only which were sometimes not even harvested from human donors [13, 28, 30]. The limitation of our study was the relatively small sample size, i.e., all the vertebrae were harvested from three donors only.

## Conclusions

Formalin fixation and freezing up to six months showed not significant effects on QCT-based BMD, trabecular bone microstructure, and FEM in intact, human vertebrae. Therefore, both preservation methods may be used in corresponding in-vitro experiments in the context of osteoporosis.
